# Large language model–based simulated patient training for heart failure palliative care communication: a pilot study

**DOI:** 10.1093/ehjdh/ztag119

**Published:** 2026-07-22

**Authors:** Risa Kishikawa, Hiroyuki Morita, Satoshi Kodera

**Affiliations:** Department of Cardiovascular Medicine, The University of Tokyo Hospital, 7-3-1 Hongo, Bunkyo-ku, Tokyo 113-8655, Japan; Department of Cardiovascular Medicine, The University of Tokyo Hospital, 7-3-1 Hongo, Bunkyo-ku, Tokyo 113-8655, Japan; Cardiovascular Biobank Research Center, International University of Health and Welfare, 4-1-26 Akasaka, Minato-ku, Tokyo 107-8402, Japan; Department of Cardiovascular Medicine, The University of Tokyo Hospital, 7-3-1 Hongo, Bunkyo-ku, Tokyo 113-8655, Japan

**Keywords:** Heart failure, Cardiovascular disease, Palliative care, Communication, Artificial intelligence, Medical education

## Abstract

**Aims:**

Heart failure (HF) palliative care communication is essential but difficult to train at scale because conventional role-play programmes require facilitators and standardized patients. Large language models (LLMs) have emerged as potential tools for scalable communication training. This pilot study aimed to evaluate the feasibility of a web-based LLM-driven communication training application and to explore its early educational signal on physicians’ self-efficacy.

**Methods and results:**

This single-arm pilot study included physicians who completed one session using a Japanese-language web-based LLM application designed to simulate patients with advanced HF and provide automated framework-based feedback. The primary outcome was change in self-efficacy scores assessed by pre- and post-session questionnaires. Ten sessions were analysed. Physicians engaged in a mean of 7.6 ± 2.0 dialogue turns. Mean response time per model output and feedback generation were approximately 3 and 17 s, respectively. Significant improvements were observed in knowledge of palliative care communication (mean difference +1.7, adjusted *P* < 0.01) and confidence in HF palliative care communication (+1.2, adjusted *P* = 0.03). Other domains showed non-significant changes.

**Conclusion:**

This pilot study demonstrated the feasibility of a web-based LLM-simulated patient system and suggested an early educational signal in physicians’ self-efficacy for HF palliative care communication. Our scalable LLM-driven communication training may complement traditional educational approaches with further evaluation in larger controlled studies.

**Clinical trial registration:**

Trial registration number: UMIN000059988

## Graphical Abstract

**Graphical Abstract:**
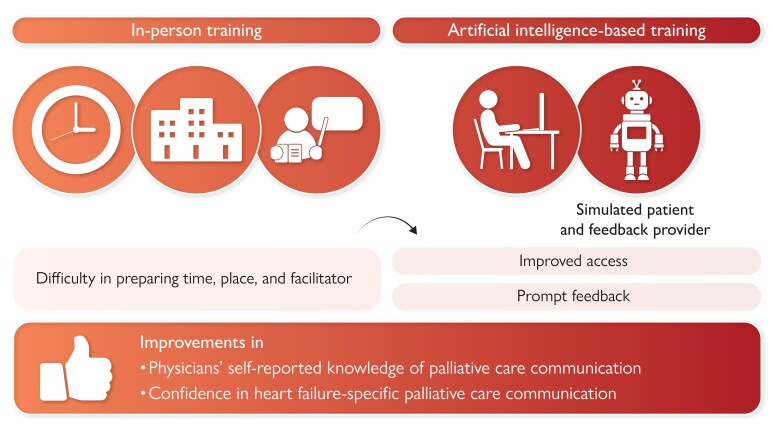

## Introduction

Heart failure (HF) is associated with high morbidity and mortality,^1^ and palliative care is increasingly recognized as an essential component of HF management because of its potential to improve patients’ quality of life.^2^ Existing training programmes often include both lectures and communication skills training through role-play.^3,4^ However, role-play training of HF palliative care communication requires the presence of standardized patients or peers and trained facilitators, and scheduling adjustments for time and location, which may limit scalability and access to the training. Additional approaches to support or facilitate learning may therefore help address training needs.

Large language models (LLMs) have enabled human-like dialogue generation and are emerging as potential educational tools. While LLM-based simulated patients have been explored in general medical education, their application to HF palliative care communication has not been reported. We developed a web-based LLM-driven communication training application designed to simulate patients with advanced HF and provide automated framework-based feedback to physicians. This pilot study aimed to evaluate feasibility of this digital training approach and to explore its early educational signal on physicians’ self-efficacy in HF palliative care communication.

## Method and results

This was a single-arm pilot pre-post feasibility study. This study was conducted in accordance with the revised Declaration of Helsinki and approved by the Institutional Review Board of the University of Tokyo (2025367NI). This trial was registered in the University Hospital Medical Information Network Clinical Trials Registry (UMIN-CTR) (UMIN000059988).

**Figure 1:**
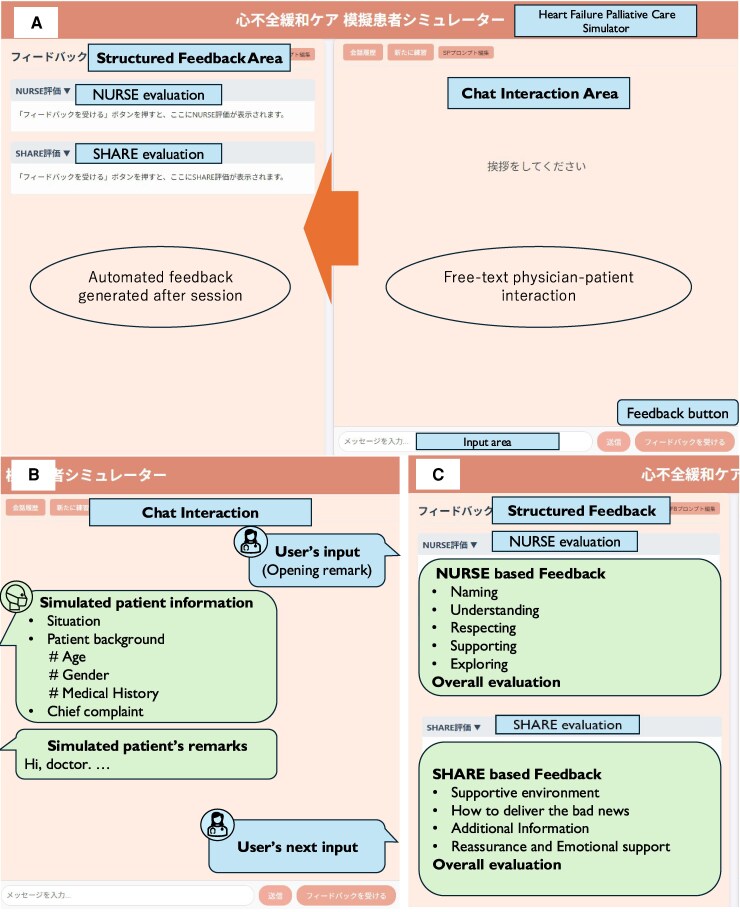
The figure illustrates how patient simulation and automated structured feedback were operationalized within the application. (*A*) Overall user interface structure of the chat-based palliative care communication simulator (with English translation). The chat interface is displayed on the right, and automated feedback is displayed on the left after the user clicks the ‘Feedback’ button. (*B*) Example of the chat-based simulation interface showing the presentation of simulated patient information and the user’s text-based interaction with the simulated patient. (*C*) Example of the automated feedback interface showing structured feedback based on the NURSE and SHARE communication frameworks.

We developed a Japanese-language web application using gpt-4o-mini-2024-07-18 (OpenAI, USA) via the Application Programming Interface for application development and deployed through a cloud-based service (Render, USA). These logs were stored using Weights & Biases (Weights & Biases, USA) for subsequent analysis. Prompt engineering was performed by a board-certified cardiologist and engineer. The prompts for LLM were composed of simulated patient and two structured feedback prompts. Representative prompt structures and feedback examples are available upon reasonable request. The simulated patient prompt includes conversation purpose and setting information, a format example for patient characteristics, few-shot examples of doctor–patient conversations. The system functioned as both a simulated patient and an automated feedback provider based on established palliative care communication frameworks, NURSE^5^ (*n*: Naming, U: Understanding, R: Respecting, S: Supporting, E: Exploring) and SHARE^6^ (S: Supportive environment, H: How to deliver the bad news, A: Additional information, RE: Reassurance and Emotional support). The patient characteristics were randomly generated by the LLM. During the dialogue, the model was instructed to act as a patient using non-technical language and to have a session with users to train their palliative care and Advance Care Planning skills.^7^ We developed a web user interface that allowed participants to view both the chat transcript and the generated feedback in one screen on a personal computer or smartphone. The chat interface, patient profile display, and feedback screen are presented in *Figure 1*. At session initiation, structured patient information was presented, and physicians conducted a free-text conversation focusing on serious illness communication and advance care planning. Physicians were instructed to press the feedback button only once after the session, and upon request, framework-based feedback was generated automatically. Simulated patient responses were streamed by character to enhance conversational realism, whereas feedback was presented as a full text block at once. Participants completed pre- and post-session questionnaires. In the questionnaires, we asked about participants’ self-assessment in communication in difficult situations, conversation skills in serious situations, response to patients’ emotions, knowledge of palliative care communication, confidence in HF palliative care communication on a 5-point Likert scale.^8^ In the post-session questionnaire, they were asked additional questions assessing their perceptions of the application.

Eleven physicians were recruited voluntarily, including seven board-certified cardiologists and seven had prior exposure to palliative training. The primary outcome was change in self-efficacy scores. Pre-post comparisons were performed using the Wilcoxon signed-rank tests with Holm correction. One session was excluded from paired analysis due to role inconsistency by the model as the model acted as a physician in the conversation, resulting in 10 physicians included in the primary analysis.

All sessions were completed without technical interruption. Physicians engaged in a mean of 7.6 ± 2.0 dialogue turns. The mean response time per model output was 3.2 ± 1.2 s, and feedback generation required 17.4 ± 4.1 s, supporting feasibility for on-demand use. Regarding usability and perception of the application, the realism of the simulated patient was rated 3.9 ± 1.0, authenticity of statements 3.5 ± 1.1, appropriateness of automated feedback 4.4 ± 0.7, ease of use 4.6 ± 0.5, and intention for continued future use 3.8 ± 1.2 on the 5-point Likert scale. Changes in self-efficacy scores are shown in *Table 1*. Improvements were observed in self-reported knowledge of palliative care communication (mean difference +1.7, adjusted *P* < 0.01) and self-reported confidence in HF palliative care communication (+1.2, adjusted *P* = 0.03). Other communication domains demonstrated small non-significant increases. Regarding communication skills, participants reported that the system helped them explicitly reflect on conversational elements that they were not always conscious of and provided confidence about their clinical approach. Regarding the application, participants generally reported positive usability, while suggesting more specific, example-based feedback and multimodal interaction as future improvements.

**Table 1:** Changes in self-efficacy scores before and after the training session

		Pre (Mean ± SD, Median[IQR])	Post (Mean ± SD, Median[IQR])	Mean difference (Post-Pre)	Holm–Bonferroni adjusted *P*
1	Communication in difficult situations	3.1 ± 1.0, 3.0[1.5]	3.4 ± 1.0, 3.5[1.0]	+0.3	1.00
2	Serious conversation skills	3.4 ± 0.8, 3.0[1.0]	3.7 ± 0.8, 3.5[1.0]	+0.3	1.00
3	Response to patients’ emotions	3.1 ± 0.6, 3.0[0.0]	3.4 ± 1.1, 4.0[1.8]	+0.3	1.00
4	Knowledge of palliative care communication	2.3 ± 0.9, 2.0[1.0]	4.0 ± 0.9, 4.0[0.8]	+1.7	<0.01
5	Confidence in HF palliative care communication	2.4 ± 0.8, 2.0[1.0]	3.6 ± 1.0, 4.0[0.8]	+1.2	0.03

Values are shown as mean ± standard deviation and median with interquartile range. Pre- and post-session scores were compared using the Wilcoxon signed-rank test. *P* values were adjusted for multiple comparisons using the Holm–Bonferroni method. Adjusted *P* values of 1.00 indicate non-significant results after Holm–Bonferroni correction. HF, heart failure; IQR, interquartile range.

## Discussion

This pilot study demonstrates the feasibility of a scalable LLM-based simulated patient platform for HF palliative care communication training and suggests an early educational signal in physician self-efficacy. Unlike traditional training formats requiring facilitators and standardized patients, the present system provided immediate structured feedback and allowed independent, on-demand practice. Participants generally perceived the system as easy to use and the generated feedback as appropriate, supporting the acceptability of this approach for communication training. The asynchronous access and facilitator-free training style may enable scalability and improve remote training potential with further evaluation in larger controlled studies.

Improvements were primarily observed in self-reported knowledge and confidence domains, suggesting that even a single text-based session may support cognitive learning and perceived readiness for communication. In medical education, receiving feedback after learning activities is considered critical for enhancing educational outcomes,^9^ which is consistent with our results. From this perspective, the quality of feedback should be guaranteed, but as our study was conducted as feasibility study, the formal expert benchmarking was lacking. Further studies should benchmark AI-generated feedback against independent expert raters. In contrast to the self-reported knowledge and confidence domains, affective communication domains changed only modestly, indicating that repeated practice or multimodal interaction may be required to influence more complex communication behaviours. Communication skills training in healthcare is considered to benefit from simulator-based education,^9^ and repeated practice may be regarded as essential.^10^ Whether similar effects can be achieved through repeated use of our model warrants further investigation. In addition, random case generation was intentionally adopted to explore real-world conversational diversity. Future versions should allow standardized case difficulty levels to evaluate the effectiveness of the platform.

The hallucination occurred during a session, in which the model acted as a physician rather than as a patient. The participant manually terminated the session, which minimized the negative impact on the training experience. Future studies should improve prompt design to reduce off-role hallucinations, and larger-scale evaluations are needed to estimate the frequency of such events before broader deployment in educational settings. Because role fidelity is essential for educational reliability, future systems should incorporate automated safeguards to detect and prevent off-role responses in real time. Limitations include the small sample size, heterogeneity in participants’ background, lack of a control group, lack of formal expert benchmark, and reliance on self-reported outcomes. Several participants had prior exposure to palliative care training, which may partly reflect the widespread dissemination of palliative care educational programmes for physicians in Japan in recent years. Thus, the findings may not be generalizable to physician populations with less prior training exposure. Accordingly, the findings should be considered exploratory and hypothesis generating and should be interpreted cautiously. Despite these limitations, scalable AI-driven communication training tools such as this platform may represent a practical and accessible complement to traditional palliative care training in cardiology.

## Conclusions

A web-based LLM-simulated patient system for HF palliative care communication was feasible and was associated with improved physician self-efficacy after a single session. This digital approach may complement existing communication training programmes and warrants further evaluation in larger controlled studies.

## Data Availability

The data underlying this article are available upon reasonable request, subject to approval by the corresponding author.

## Lead author biography

Dr. Risa Kishikawa is a cardiologist at the University of Tokyo Hospital in Tokyo, Japan. She graduated from the Faculty of Medicine at the University of Tokyo in 2015. After completing her clinical training and cardiology residency at Showa General Hospital in Tokyo, she began her research in medical AI at the Graduate School of Medicine of the University of Tokyo, obtaining her PhD in Medicine in 2024. She conducts research with the goal of using AI to diagnose cardiovascular diseases and to aid in palliative care.

## References

[1] Tromp J, Bamadhaj S, Cleland JGF, Angermann CE, Dahlstrom U, Ouwerkerk W, et al Post-discharge prognosis of patients admitted to hospital for heart failure by world region, and national level of income and income disparity (REPORT-HF): a cohort study. Lancet Glob Health 2020;8:e411–e422. 32087174 10.1016/S2214-109X(20)30004-8

[2] Gelfman LP, Blum M, Ogunniyi MO, McIlvennan CK, Kavalieratos D, Allen LA. Palliative care across the spectrum of heart failure. JACC Heart Fail 2024;12:973–989. 38456852 10.1016/j.jchf.2024.01.010

[3] Li WW, Chhabra J, Singh S. Palliative care education and its effectiveness: a systematic review. Public Health 2021;194:96–108. 33873061 10.1016/j.puhe.2021.02.033

[4] Shibata T, Oishi S, Mizuno A, Ohmori T, Okamura T, Kashiwagi H, et al Evaluation of the effectiveness of the physician education program on primary palliative care in heart failure. PLoS One 2022;17:e0263523. 35120191 10.1371/journal.pone.0263523 PMC8815870

[5] Back AL, Arnold RM, Baile WF, Tulsky JA, Fryer-Edwards K. Approaching difficult communication tasks in oncology. CA Cancer J Clin 2005;55:164–177. 15890639 10.3322/canjclin.55.3.164

[6] Fujimori M, Shirai Y, Asai M, Akizuki N, Katsumata N, Kubota K, et al Development and preliminary evaluation of communication skills training program for oncologists based on patient preferences for communicating bad news. Palliat Support Care 2014;12:379–386. 24182602 10.1017/S147895151300031X

[7] Millstein LS, Rosenblatt P, Bellin MH, Whitney L, Eveland SR, Lee MC, et al Advance care planning and communication skills improve after an interprofessional team simulation with standardized patients. Palliat Med Rep 2022;3:123–131. 36059907 10.1089/pmr.2021.0086 PMC9438443

[8] Berlacher K, Arnold RM, Reitschuler-Cross E, Teuteberg J, Teuteberg W. The impact of communication skills training on cardiology fellows’ and attending physicians’ perceived comfort with difficult conversations. J Palliat Med 2017;20:767–769. 28437212 10.1089/jpm.2016.0509

[9] Motola I, Devine LA, Chung HS, Sullivan JE, Issenberg SB. Simulation in healthcare education: a best evidence practical guide. AMEE guide No. 82. Med Teach 2013;35:e1511–e1530. 23941678 10.3109/0142159X.2013.818632

[10] Niglio de Figueiredo M, Krippeit L, Ihorst G, Sattel H, Bylund CL, Joos A, et al ComOn-coaching: the effect of a varied number of coaching sessions on transfer into clinical practice following communication skills training in oncology: results of a randomized controlled trial. PLoS One 2018;13:e0205315. 30289905 10.1371/journal.pone.0205315 PMC6173449

